# ﻿The History and introduction of the Daurian Lily *Liliumpensylvanicum* and the new combination *L.pensylvanicum* var. *alpinum* (Liliaceae)

**DOI:** 10.3897/phytokeys.236.111741

**Published:** 2023-12-22

**Authors:** James A. Compton, Andrej K. Sytin

**Affiliations:** 1 Spilsbury Farm, Salisbury, SP36RU, UK Spilsbury Farm Salisbury United Kingdom; 2 Komarov Botanical Institute of the Russian Academy of Sciences, Prof. Popov Street 2, St. Petersburg 197376, Russia Komarov Botanical Institute of the Russian Academy of Sciences St. Petersburg Russia

**Keywords:** Amman, Catesby, Collinson, Demidov, Dillwyn, Gmelin, Heydenreich, Ker Gawler, *Liliumdauricum*, *Liliumpensylvanicum* var. *alpinum*, Messerschmidt, nomenclature, Steller, typification

## Abstract

Manuscripts in the Archives of the Academy of Sciences in St. Petersburg reveal the first recorded observations and introductions of *Liliumpensylvanicum* Ker-Gawl. from Siberia to European Russia. The naming of *Liliumpensylvanicum* and its attempted renaming to *L.dauricum* Ker-Gawl. is fully outlined. Lectotypes are designated here for the names *Liliumpseudodahuricum* M.Fedoss. & S.Fedoss., L.dauricumvar.alpinum N.I.Kuznetsov and L.pensylvanicumf.praecox Vrishcz. The new combination L.pensylvanicumvar.alpinum (N.I.Kuznetsov) J.Compton & Sytin is made and a key is provided to the varieties of *L.pensylvanicum*.

## ﻿Introduction

Species of the genus *Lilium* L. with scattered leaves, upright-facing, cup or bowl-shaped flowers and tepals that narrow to a basal claw are found in North America, Europe and east Asia. *Liliumcatesbaei* Walter and *L.philadelphicum* L., the only two North American species with that morphology, are endemic to that continent. *Liliumbulbiferum* L. is European and *L.concolor* Salisb., *L.pensylvanicum* Ker-Gawl. and *L.maculatum* Thunb. occur across east Asia and Japan. The first of the upright, bowl-flowered, Asian species of *Lilium* to be introduced to western Europe was almost certainly *L.pensylvanicum*, described with a pre-Linnaean phrase name by Johann Georg Gmelin (1709–1755) from Siberia. Gmelin cited a paper by the Prussian physician, chemist and mineralogist Johann Friedrich Henckel (1678–1744) discussing a collection of the lily by the metallurgist Johann Gottfried Heydenreich. This species was collected by Heydenreich from Siberia in the late 1720s and introduced into what was then Saxony shortly thereafter (Henckel 1733: 354; Gmelin 1747: 41).

Due to their morphological similarities, all the above-mentioned *Lilium* species have been considered to be closely related (e.g. Baker (1871); Wallace (1873)). Phylogenetic analyses of molecular sequence data from nuclear DNA ITS, however, (e.g. Du et al. (2014); Huang et al. (2018); Givnish et al. (2020)), as well as from both cpDNA and nuclear DNA (Watanabe et al. 2021), have shown that the two North American species *L.catesbaei* and *L.philadelphicum* are more distantly related to the others and belong in Liliumsect.Pseudolirium (Endl.) Spach (Spach 1846: 277) typified on *L.catesbaei* (Wilson 1925: 50), whereas *L.bulbiferum*, *L.concolor*, *L.maculatum* and *L.pensylvanicum* all belong in sect. Sinomartagon H.F.Comber typified on *L.davidii* Duch. (Comber 1949: 101). *Liliumconcolor*, known as the morning star lily, has terete, glabrous stems, rather than ribbed or papillose-scarious stems in *L.bulbiferum*, *L.maculatum* and *L.pensylvanicum*, the flowers are stellate in appearance as opposed to bowl-shaped and the tepals are shorter, ca. 3–4 cm as opposed to 5–9 cm long in the other species. Although native in China, Japan, Korea and Russian Far East, *L.concolor* is not part of the discussion in this paper.

There is no doubt that the closest relative of *L.pensylvanicum* is *L.maculatum* from Honshu, Japan. This species, is distinguished from *L.pensylvanicum* by its lacking the floccose pubescence on its stems and leaf axils. The stems of *L.maculatum* are instead papillose and scarious and the germination mode is epigeal as opposed to hypogeal (Hayashi 2016: 112). Recent molecular data also confirm the segregation of these species (Watanabe et al. 2021: 192, 193).

The two North American species are distinguished from *L.bulbiferum*, *L.maculatum* and *L.pensylvanicum* by their glabrous stems and leaves and narrower tepals. In *L.catesbaei*, these are 1.2–1.9 cm wide (Skinner 2003: 179), whereas in *L.philadelphicum*, they are 2–3.2 cm wide (Skinner 2003: 180). The American species also have longer claw-like tepal bases which partly enclose the nectar guides near the base of the tepal lamina. *Liliumphiladelphicum* frequently has at least one whorl of leaves subtending the inflorescence and characteristically long seed capsules which attain 2.2–7.7 cm in length (Skinner 2003: 180).

The European species *L.bulbiferum* was known to very early writers and was frequently discussed and illustrated in several works across Europe, for example by Dodoens (1553: 237; 1583: 198); L’Obel (1576: 84); Clusius (1583: 139–141) and Caspar Bauhin (1623: 75–76). Bauhin’s *Pinax* was much cited by subsequent botanists who usually indicated reference to his work simply by citing his initials. *Liliumbulbiferum* was so named for the production of small bulbils in the leaf axils along the inflorescence axis; however, plants lacking such axillary bulbils were also included within the circumscription of this species (Linnaeus 1753: 302). Those plants which lacked bulbils were later segregated as *L.croceum* Chaix (Villars 1786: 322). These two taxa which are restricted to Europe and have more or less glabrous leaves and stems are undoubtedly closely related to *L.pensylvanicum*, but are not the focus of this paper.

**Figure 1.**
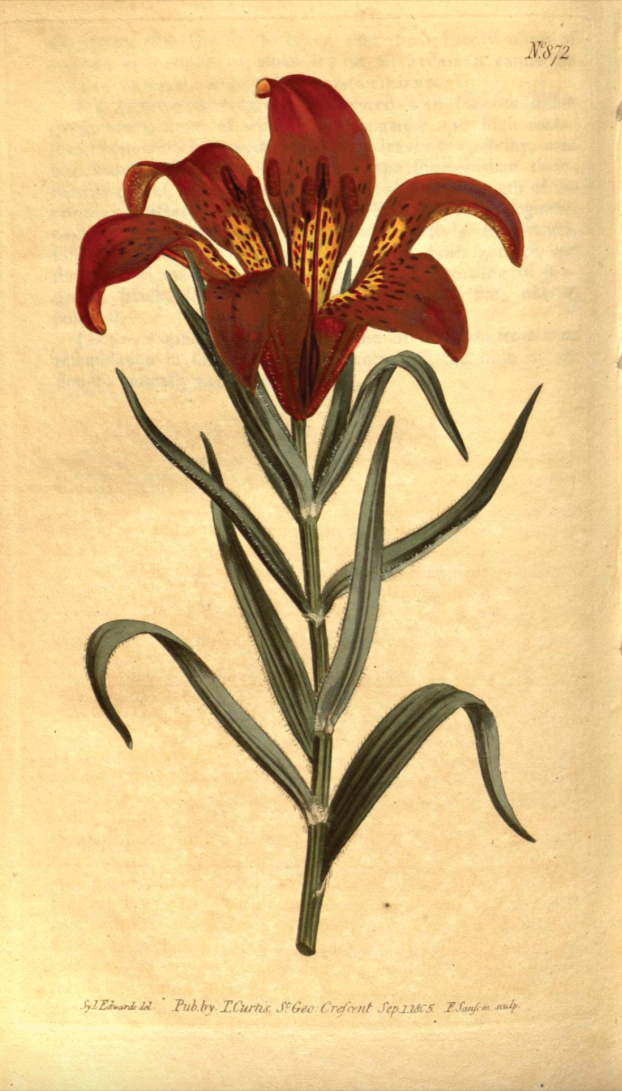
Sydenham Edwards illustration of *Liliumpensylvanicum* in Curtis’s Botanical Magazine 22 t. 872 (1805).

This paper looks closely at the history and nomenclature of *L.pensylvanicum* (see Fig. 1) along with notes on its introduction from Siberia and references to the people who were associated with it. The mistaken belief that its origin was in North America is carefully scrutinised along with its journey from Dauria in east Siberia to the herbaria and gardens of Imperial Russia and thence to England. Literary, archival and illustrated materials were used to track its route from Siberia to northern Europe. The attempt to rename it *L.dauricum* Ker Gawl. (Ker Gawler 1809b) is also fully examined. A proposal to conserve *L.dauricum* against *L.pensylvanicum* by Y-D. Gao in Taxon 70: 1139–1140 (2021), has been rejected (Taxon 72: 908–922 (2023)), meaning that *L.pensylvanicum* is maintained as the correct name for the Daurian lily.

## ﻿Messerschmidt’s Siberia Expedition 1720–1727

The first collector in the Russian service to explore the natural history of Siberia was Daniel Gottlieb Messerschmidt (1685–1735). Originally from Danzig [Gdansk], Messerschmidt, having been introduced to Tsar Peter I of Russia in 1716 by Johann Philipp Breyne, had been asked by him to explore the nature of the great majesty of the Russian Empire. Two years later, he was tasked to collect medicinal and other plants on an expedition that he undertook from 1720 to 1727 across Russia to Siberia (Sytin 2004; Koroloff 2014).

Messerschmidt sent a large box full of seeds and an herbarium back to the Aptekarskiy Ogorod or Apothecary Gardens in St. Petersburg founded by Tsar Peter in 1714 on Aptekarskiy Ostrov [Aptekarsky Island] (Koroloff 2014: 126). This site which now houses the Komarov Botanical Institute, would have been the main botanical collection point for new introductions until the founding of the botanic garden of the Academy of Science in the mid-1730s (Sokoloff et al. 2002: 147). The seeds would have been tended by the director of the garden Johann Christian Buxbaum (1693–1730). Evidence of the lily’s cultivation in the garden, however, does not exist, in large part due to a disastrous fire that occurred in the Medical Chancellery in 1737 which destroyed the entire archive of the Apothecary Garden (Koroloff 2014: 138).

While in Siberia, in September 1724, Messerschmidt’s expeditionary detachment travelled from Chita to Irkutsk skirting around the southern end of the enormous Lake Baikal. During a severe snowstorm, they lost their bearings and went too far south. They ended up between the Onon and Ulz Rivers in Mongolian China where their arrival was brought to the attention of the local Chinese authorities. Messerschmidt and his retinue had to remain in Selenginsk throughout the winter months until March 1725 when they were able to continue to Udinsk (now Ulan Ude). During that period, Messerschmidt wrote a dictionary of the Mongolian and Tangut language with assistance from several local teachers. In that list, he cited as entry 67b.8 “Ssaranà Liliumpurpurocroceum feüer Lilien” with the word Ssaranà also written in Khalkha Mongolian (Sizova et al. 2022: 98). Messerschmidt’s citation of Bauhin’s “Liliumpurpurocroceum” (Bauhin 1623: 77) and the German “feüer lilien”, meaning fire lily, both terms often used for the morphologically similar *L.bulbiferum*, indicate that he had already encountered *L.pensylvanicum* in Siberia.

Further evidence of Messerschmidt’s finding of the Daurian lily is found in his unpublished journal: "*Pinacis simplicium regnum vegetabile seu plantae medicae*" in which 1290 medicinal and useful plants are included (Messerschmidt 1720–1727). He listed as number 502: *Lilium purpuro-croceum majus* along with a note: “In Russia in exterorum quorundam botanophilon hortis Moscuae. Ejus species minor in Sibiria, in Chatanga, Lena, Angara, Ingoda, Schilka, Argun et Onon fluviorum montanis et apricis, sat abunde” [In Russia, it is found in the Moscow gardens of those who love botany. Its smaller Siberian species is found abundantly in sunny places and in the mountains alongside the rivers Khatanga, Lena, Angara, Ingoda, Shilka, Argun and Onon].

## ﻿Gmelin and the Great Northern Expedition 1733–1743

Johann Georg Gmelin (1709–1755), born in Tübingen, moved to St. Petersburg in 1727 where he assisted in the Kunstkammer, the first public Museum in Russia, which had been founded by Tsar Peter I in 1704. Gmelin became an adjunct of the Academia Scientiarum Imperialis Petropolitanae [Imperial St. Petersburg Academy] in 1730 and was employed by the Academy from 1731 as Professor of chemistry and natural history (Sokoloff et al. 2002: 154). The Second Kamchatka Expedition also known as the Great Northern Expedition, one of the largest exploration enterprises in human history, took place from 1733 to 1743 and was instigated under the aegis of Tsarina Anna Ivanovna. The distance covered was phenomenal, from St. Petersburg to Okhotsk on the east coast is more than 3400 miles or 5500 km. Gmelin, as one of three professors sent out by the Academy, led the Natural History deputation, accompanied by his assistant Stepan Petrovich Krascheninnikov (1711–1755). Their mission was to join the Danish sailor in the Russian service, Vitus Jonassen Bering in order to ascertain if Kamchatka and Alaska were linked and to collect and record all forms of natural and cultural history along the way.

During the third year of the expedition in November 1736, a drastic fire broke out in Yakutsk in central Siberia which burnt Gmelin’s collections, drawings and part of his library. As a result, he had to remain in the Yakutian part of Siberia to gather new collections and this prevented him from ever reaching the Pacific (Sokoloff et al. 2002: 158). In 1737, Gmelin and the Academy Professor of ethnology and natural history, Gerhard Friedrich Müller (1705–1783) sent Krascheninnikov on to Okhotsk on the Pacific coast and then to the Kamchatka Peninsula where he remained collecting natural history material for the next four years.

## ﻿Steller an adjunct of the Academy

Georg Wilhelm Steller (1709–1746), originally from Bad Windsheim, Bavaria, arrived in St. Petersburg in November 1734. He was introduced to the Swiss botanist Johann Amman (1707–1741), who was, at that time, a recently appointed Assistant Director of the Kunstkammer or Cabinet of Curiosities, the first public museum in Russia, officially the Museum Imperialis Petropolitani. Steller also met Daniel Messerschmidt shortly before the latter’s death in 1735. Steller then subsequently married his widow Birgitta and was hired as an adjunct of the Academy in February 1737 specifically to join the Great Northern Expedition.

Ernest Wilson, in the introduction to his monographic book The Lilies of Eastern Asia, stated that Steller had discovered the Daurian lily in 1737 in the region of the river Lena (Wilson 1925: 7). Steller, in fact, only set off east from Moscow in March 1738 for Tobolsk (Engel and Willmore 2020: 180). He left his wife behind and was accompanied by the illustrator Johann-Cornelis Dekker. He eventually met the Academy Professors Gmelin and Müller in the Siberian city of Yeniseysk on 7 December 1738 (Krascheninnikov 1764: v; Engel and Willmore 2020: 180). It is highly probable that Wilson had confused Gmelin’s collection (mentioned below) with Steller’s.

Steller however, collected plants of the lily in Siberia later and sent back plants or seeds to Amman in St. Petersburg. In a letter that Steller wrote on 24 December 1739 from Irkutsk to Johann Daniel Schumacher, Secretary of the Russian Academy of Sciences and Director of the Academy’s Library, he mentions sending back to Amman his collection of seeds and plants (Engel and Willmore 2020: 186). Amman had been appointed the first director of the Academy’s new botanic garden in 1735 (Sokoloff et al. 2002: 151). This despatch to St. Petersburg apparently contained six boxes of herbarium specimens, seeds, plants and other natural history items. The Russian authorities temporarily impounded the collection for some time in Yeniseysk (Sokoloff et al. 2002: 155). Amman, however, received several boxes of plants from Siberia sent by Steller on 30 May 1740 (Sytin 2022: 190).

Steller wrote about his encounters with a Siberian lily. On 18 June 1740, he wrote in his journal from the Lena River near Yakutsk “*Walking back I noticed a wild growing blood red lily which Dr Gmelin had noticed on the Irtysh River and I had found among the dried plants Mr Rosing the pharmacist in Kyakhta had collected around the Kyakhta River*” (Engel and Willmore 2020: 116). On 3 July 1740, he wrote from near the Amga River, the largest tributary of the Aldan: “*Today I noticed a wild Iris, northern Indian paint-brush with white and red flowers, a cinquefoil with long runners, the wild blood-red Siberian lily and a species of lousewort*” (Engel and Willmore 2020: 126). It is possible that he was referring to *L.pensylvanicum*, but equally to *L.pumilum*.

Steller was given instructions by Gmelin in 1739 to continue his journey onwards to Kamchatka. He was accompanied by the archaeology student Aleksei Petrovich Gorlanov and the painter and scientific illustrator Johann Christian Berckhan (1709–1751). On their onward journey south-east from Yeniseysk towards Lake Baikal, Steller and his companions were delayed for almost a year in Irkutsk near the western shore of Lake Baikal. They explored the flora, minerals and fauna around Barguzin during the summer for six weeks on the eastern shores of Lake Baikal until September 1739 (Engel and Willmore 2020: 180). During that time, Steller wrote his unpublished manuscript Flora Irkutensis in which he listed 1152 plant names including as number 495 the name *Lilium minii colore cruentum*, a reference to the Daurian lily (see below). They continued first to Kyakhta on the Siberia-Mongolian border (then part of China), then along the River Lena to Yakutsk in March 1740, then overland to Okhotsk where they met up with Vitus Bering on 14 August 1740 (Engel and Willmore 2020: 181). Berckhan, Gorlanov and Steller then joined up with the Academy student Stepan Krascheninnikov who was already in Kamchatka.

## ﻿Gmelin’s Flora Sibirica and Heydenreich

Gmelin published his finding of the upright Daurian lily in the first volume of Flora Sibirica (Gmelin 1747: 41–42) as “Lirium number 8”. He described the plant using Linnaeus’s phrase name for *Liliumbulbiferum*: “foliis sparsis, corollis campanulatis, erectis, intus scabris, Linn” (Gmelin 1747: 41). Gmelin stated that the phrase name was taken directly from Linnaeus’s Hortus Cliffortianus (Linnaeus 1738: 120). This same phrase name was later used by Linnaeus in the validating description of *L.bulbiferum* (Linnaeus 1753: 302). Linnaeus had catalogued the morphologically similar European *L.bulbiferum* growing in the garden of the rich Dutch East India Company merchant George Clifford III at de Hartekamp in the Netherlands (Linnaeus 1738: 120). Linnaeus later, in his protologue for *L.bulbiferum*, also included the reference Gmel. Sibir. 1 p. 41 (Linnaeus 1753: 302) believing that the Siberian plant was in fact *L.bulbiferum*. Both Linnaeus and Gmelin each included a reference to Adriaan van Royen’s Flora Leydensis prodromus (van Royen 1740: 31). After his work for George Clifford, Linnaeus went to Leiden in 1737 where he helped the Leiden garden’s Director, van Royen, to compile his volume on the plants cultivated there. Clearly both Gmelin and Linnaeus believed they were dealing with a single widely dispersed species occurring in both Europe and east Asia.

In the dissertation presented to Linnaeus on 15 May 1766 with the title: Necessitas Historiae Naturalis Rossiae by the Russian student of metallurgy at Moscow University, Alexander Matwejewitsch von Karamyschew, he referred to the immense value of Gmelin’s Flora Sibirica (Karamyschew 1769: 443). In the list of useful plants from Siberia under the heading ‘Flora Sibirica’, Karamyschew lists under *Hexandria* four lily names: number 59, *Liliumbulbiferum*; 60, *L.pomponium*; 61, *L.martagon* and 62, *L.kamschat*. (Karamyschew 1769: 461). His number 59 was without doubt *L.pensylvanicum*, but was categorically recognised by Linnaeus as *L.bulbiferum*.

In his Flora Sibirica treatment, Gmelin divided this upright-flowering Siberian *Lilium* species into the modern-day equivalent of two varieties: 1. *Folialatioribus*, plants with broad leaves and 2. *Foliaangustioribus*, narrow-leaved plants, the latter divided further into the equivalent of two formae: α. *Floreminiato*, plants with red flowers and β. *Floreluteo*, yellow-flowered plants (Gmelin 1747: 41). Gmelin included additional information about these taxa, stating that they were found everywhere between the Yenisey River eastwards to Okhotsk on the Pacific coast, especially in fields and near rivers and streams. He added that, where they grew in the wild, they frequently had only one, two or three flowers and the leaves often had marginal hairs. Gmelin further stated that the var. 2 α with narrow leaves and red flowers, was most commonly found in regions around the Rivers Lena, Aldan, Maya and Yudoma and the var. 1 with broad leaves was found throughout. Gmelin went on to state that, apart from their height and the width of their leaves, var. 1 and var. 2 were very similar. He added that the narrow-leaved and yellow-flowered var. 2 β was collected by his adjunct Steller who had written to him to say that he had found it growing below the City of Yakutsk near the Lena and Aldan Rivers and that the leaves were whitish underneath, whereas he, i.e. Gmelin, had always observed that those of the red-flowered forma α were green on both sides (Gmelin 1747: 42).

Gmelin‘s next entry “Lirium number 9” was another species of *Lilium* found in Siberia which he described as “radice tunicata, foliis sparsis, floribus reflexis, corollis revolutis” (Gmelin 1747: 42). He added “Lilium reflexum montanum, humile, angustifolium, aurantium, Sarana Mungulis in Dauria Mess. Amm. Ruth. No. 138” and “Bauhin Lilio byzantine miniato”, a direct reference to the scarlet-flowered European *Liliumchalcedonicum* L. to which he compared the species as being very similar (Gmelin 1747: 43). It was almost certain that this Siberian scarlet turkscap species was listed without any description in 1812 as *L.tenuifolium* on page eight in the Catalogue of plants growing in the garden of Count Alexis Razoumoffsky (1748–1822) at Gorenki Palace, Balashikha near Moscow. It was, however, first validly described and depicted by the famous botanical illustrator Pierre-Joseph Redouté as *L.pumilum* Redouté in Paris later that same year.

In addition to the information on the Daurian lily number 8, Gmelin added three references to his broad-leaved var 1: *Liliumpurpureo-croceummaius* (Bauhin 1623: 76), *Liliumpurpureummaius* (Dodoens 1583: 198) and the query “? *Lilium floris rubro-lutei*, *Tangunensibus Sarana polevvaga appellatum* Act. Nat. Cur vol. iii p. 355”, followed by the comment “Videtur. Auctor relationis populum nominans Tungusos intelligit. Nomen Tungusis adscriptum Russicum est, legendum sarana polevvaja (Lilium campestre) [Apparently, the author of the report understands that the people who gave this name are the Tungus. The Tungusic name is attributed in Russian as sarana polevvaja (лилия полевая or lily of the field)] (Gmelin 1747: 41). The first two of these references undoubtedly refer to the Daurian lily unequivocally as the European *L.bulbiferum*. The third one, which Gmelin included with a question mark, was the reference Act. Nat. iii p. 355. This refers to a short paper presented by the Prussian physician, mineralogist and metallurgist Johann Henckel in "Acta physico-medica Academiae Caesareae Leopoldino Carolinae Naturae" vol. 3: 355 (1733). Henckel’s paper, dated Dresden 28 March 1732, was entitled ‘Plantis Sinensium, ad confinia Siberiae australis nuper observantis’ [Chinese plants recently observed on the borders of southern Siberia]. Henckel stated that his great friend and fellow metallurgist Johann Gottfried Heydenreich had recently returned [to Saxony] with 37 collections of seeds that he had collected along the Chinese – Siberian border. In fact, this was the border with modern-day Mongolia. Heydenreich had presented these to the King (Henckel 1733: 354). It is difficult to ascertain to which king Henckel was referring, but it was probably to Augustus II (1670–1733) known as Augustus the Strong, King of Poland and who was also Freidrich Augustus I, Elector of Saxony.

Heydenreich [also Heidenreich] was a mining specialist from Saxony who was initially employed to work for Tsar Peter I’s recently formed Berg-kollegia or Collegium of Mining in 1722 alongside Vilim Ivanovich de Gennin (né Georg Wilhelm Henning). In May 1728, he was sent to examine silver deposits near Nerchinsk in southern Siberia as a chief technical specialist. He returned to Saxony in 1730 (N. Koparenov in Enzyklopädie der Russlanddeutschen https://enc.rusdeutsch.eu/articles/5560 accessed 2 Jan 2023).

Four of Heydenreich’s collections were of lilies of which number eleven on the list stated simply “*Lilium* cujus folia Mongalenses coquunt cum carnibus [*Lilium* the leaves of which the Mongolians cook with meat]. Number 12 on the list included the information “*Lilium, floris rubro-lutei, Tangunensibus Sarana polevvaga appellatum: radicem siccatam loco pannis edunt, partier et carnibus coquendis addunt*” [the lily with red-yellow flowers is called by the Tanguts sarana polevvaga: they eat the dried roots instead of bread and as an addition when they cook meats] (Henckel 1733: 355). There is a potential confusion here between the Tungusic peoples [the Evenki] mentioned by Gmelin, who inhabited the lands between the Yenissei River and Lake Baikal in Siberia and the Tanguts of northern China. Although the name sarana polevvaga may have been that used by the Tungus, it was from the Tangut/Mongolian Region that Heydenreich had collected his seeds. Gmelin, in fact, also discussed the virtues of sarana, adding that it was known under that name by the Tungus, Buryats and Yakuts where it was boiled up with milk. He added that the Russians either referred to it as “toothed” because of the scales on the bulbs or “of the plains” due to its habitat and that it was also called by the Yakuts “Korun” (Gmelin 1747: 42).

Gmelin believed that his broad-leaved variant of the lily was probably the same as that described earlier by Henckel from seeds collected in Siberia by Heydenreich. There is no record of where or, indeed, by whom the lily was cultivated when it arrived in Saxony, but it could have been to Freiberg where Henckel was living as a physician and was made Councillor of Mining from 1732. Henckel stated that the plant was given to the King, perhaps this plant was cultivated at the royal palace of Dresden Castle or in the garden of the summer palace at Schloss Pillnitz on the River Elbe outside Dresden; however, this is conjecture.

**Figure 2.**
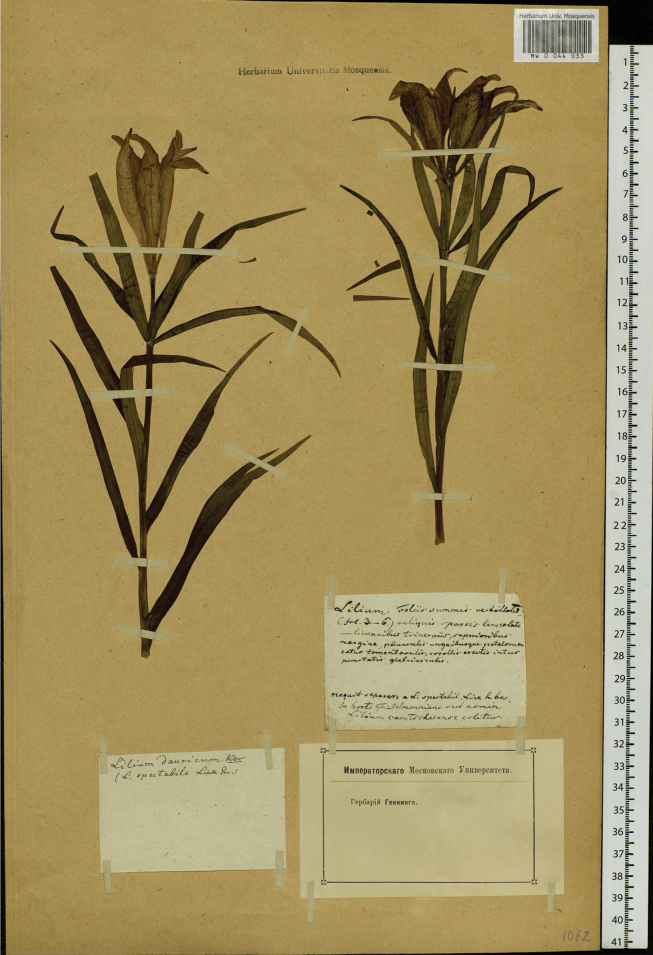
Moscow University specimen MW0044033 of *L.pensylvanicum* bearing ‘In horto Fintelmanniano’ label.

There is an interesting herbarium specimen of *L.pensylvanicum* in the Herbarium of Lomonosov Moscow State University [MW0044033] with a label for the Imperial Moscow University and Herbarium Genning (Fig. 2). The Imperial Moscow University was founded in 1755 by Tsarina Elizaveta Petrovna. During the French invasion of Russia in 1812 under Napoleon, the University of Moscow building was razed to the ground, but many natural history specimens had already been evacuated to Nizhny Novgorod 270 miles (440 km) to the east. On this Moscow sheet, there is another much older label with the annotation in ink “nequit seperare a L.spectabile Link h. Berol. in horto Fintelmanniano sub nomen Liliumcamchatkense colitur” [this cannot be separated from *L.spectabile* Link in the Hortus Berolinensis and is cultivated in the Fintelmann Garden under the name *Liliumcamchatkense*]. The reference to the garden of Fintelmann is likely to be that of one of the descendants of Heinrich Fintelmann (d. 1733) whose progeny were gärtners and hofgärtners at Charlottenburg Palace in Berlin. The reference to *L.spectabile* Link is to a superfluous synonym of *L.pensylvanicum*. A possible cultivator of the plant, therefore, is Joachim Anton Ferdinand Fintelmann (1774–1863), hofgärtner at Charlottenburg, who may have sent the specimen to Moscow from the royal garden in Berlin. Again, this can only be conjecture.

Gmelin (1747: 41) also included two references to his narrow-leaved var. 2: *Liliumpurpureo-croceumminus* (Bauhin 1623: 77) and *Liliumpurpureumminus* (Dodoens 1583: 198). These references also both refer equally to the European *L.bulbiferum*, but in this context to the lily’s counterpart in Siberia which, according to him, in one of the variants, is smaller and has narrower leaves than those described in var. 1. No specimen has been found to confirm this variety’s identity, but Gmelin could have been referring to what was later described as L.dauricumvar.alpinum N.I.Kuznetsov.

## ﻿The Daurian lily’s first records and illustrations

Over a decade after Messerschmidt’s death in 1735, records of the dispatch of *L.pensylvanicum* as a living plant from Siberia to St. Petersburg are to be found in Amman’s published and unpublished catalogues. These were a survey of the plants grown in the garden of the Academy of Science on Vasilevsky Ostrov [Vasilevsky Island] prepared shortly before his death. *Liliumpensylvanicum* is found in Amman’s unpublished catalogue as number 863 under the name: “*Lilium minii colore cruentum*”, citing Gmelin as the collector and the River Lena as its place of origin (Amman 1739–1740: 41).

**Figure 3.**
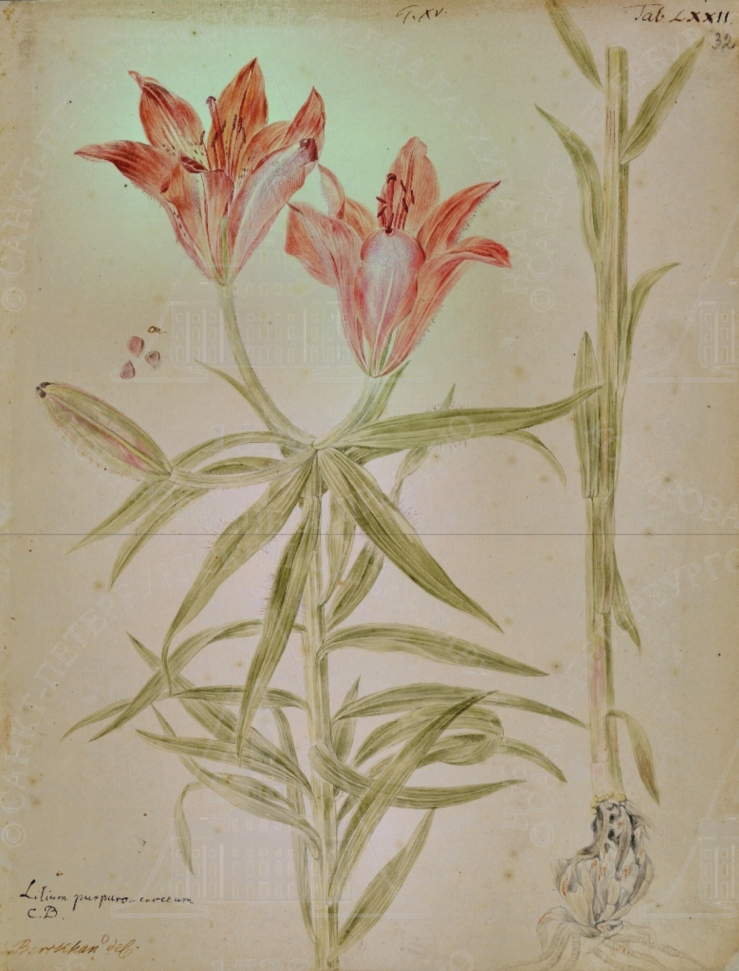
Watercolour of *L.pensylvanicum* by Johann Christian Berckhan t. LXXII ca. 1736-1737.

One of the many people accompanying Gmelin on the Great Northern Expedition was the scientific illustrator Johann Christian Berckhan. In the archives of the Academy of Sciences in St Petersburg is a watercolour of *L.pensylvanicum* painted by Berckhan t. LXXII online as http://ranar.spb.ru/rus/vystavki/id/710/ (Fig. 3). The painting is annotated “Berkhan del.” in his hand and in ink above in Gmelin’s hand “Lilium purpuro-croceum C.B.”, the initials C. B. referring to the name Lilium purpuro-croceum in Caspar Bauhin’s *Pinax*, the name of the morphologically similar *L.bulbiferum* (Bauhin 1623: 76). According to Gmelin’s own unpublished notes: Index vegetablium ad Lenam fluvium nascentium, annis 1736–1737 (or Index Lenensis) in the archives of the Academy, this painting was made by Berckhan near the River Lena during the summer of 1736 or 1737 and numbered with Roman numerals (Gmelin 1736–1737). In Gmelin’s MS, this species is described under number 443 on sheet 32v-33 as “Lilium minii colore cruentum Licet cum Lilio purpuro-croceo majore C.B.P”. The draft MS later had added to it “Tab. LXXII” (see Fig. 3) corresponding to the number on Berckhan’s watercolour. Gmelin added in his MS with respect to the location and habit of the plant in the watercolour: “Floret sub medium Junii ad omnem Lenam ad Schiganensia usque hibernacula” [It blooms from the middle of June and grows throughout the Lena to Zhigansk Districts until winter]. Berckhan did not return to St. Petersburg until 1746, several years after his colleagues had all returned and was, therefore, not involved in the preparation of the 297 illustrations for the four volumes of Gmelin’s Flora Sibirica. No illustration of the lily featured in that work.

The drawing t. 72 by Berckhan was copied by Georg Steller who wrote in his unpublished Flora Irkutensis: “495. Lilium minii colore cruentum, cuius Iconem procuravit D. D. Gmelin vidi Tab. LXXII” [I have seen the flame-blood coloured lily, whose icon t. LXXII the illustrious Dr Gmelin procured] (Steller 1739a: 90v, 91). Steller then lists references to L’Obel (1581: 169, t. 208) and Bauhin (1623: 78), although, both these references are to the red-flowered turkscap European species *L.chalcedonicum*. Steller continues with additional information about the species “Planta haec perennis sub initium Junii in montosis apricis ad Angaram et circum lacum Baikal abunde florens” [This perennial plant blooms profusely at the beginning of June in the sunny mountains by the River Angara and around Lake Baikal] (Steller 1739a: 104, t. 91–91v.). He alludes to its production of underground stolons [….] “facile e bulbo veluti vagina extrahitur, qua parte e bulbo prominent terrae legitur, fibras albas tenues emittit, terrae inhaerentes, iisque prossus similes, quae bulbo inferius ad haerescunt [….it sends out thin prostrate stems which adhere to the earth and which remain attached to the bulb]. Finally, Steller alludes to the edible qualities of the bulb of this species “Russis et Tataris accredit Сарана, quam Russi addito distinguere solent a priori, quoad radicem Glava, in cibo utrorumque” [The Russians and the Tartars both value Sarana, the Russians distinguish their food from that of the Tartars by the addition of the root Glava]. Steller’s still unpublished Flora Irkutensis with 1152 plant descriptions was completed by December 1739 (Engel and Willmore 2020: 180).

Steller also produced a seed list from his collecting activities in Siberia, from around Barguzin in Buryatia, Irkutsk and Lake Baikal (Steller 1739b). On page 15 of this list, Steller included as number 200 “Semen Lilii Cat. Pl. no. 495. In tribus chartis Horum liliorum etiam bulbi siccati-mitturitur” [Seed of lilies in the Catalogue of Plants. number 495. In the three papers of these lilies will also be sent the dried bulbs]. The number 495 was the same number for *L.pensylvanicum* as that which he had used in his Flora Irkutensis MS.

## ﻿Amman the Academy’s Garden Director

Gmelin, his student Krascheninnikov and the Academy adjunct Steller all sent seeds to the Swiss Johann Amman (1707–1741), the Academy Botanic Garden’s first Director and Professor of Botany (Koroloff 2014: 134; Sytin 2022: 171). During his earlier time in England as an assistant to Sir Hans Sloane, Amman had forged contacts with several eminent botanical and horticultural figures. Amongst these were Peter Collinson in Peckham and most significantly Mark Catesby for whom he was a subscriber to his Natural History of Carolina (Catesby 1731–1743) and to whom he donated and inscribed his name in a copy of his published catalogue of the St. Petersburg Academy Garden, *Stirpiumrariorum* (Amman 1739). This copy is in the Smithsonian Institution Library.

After Müller’s visit to London, in 1733, Amman joined the Academy of Sciences in St. Petersburg. Müller, meanwhile, was to leave St. Petersburg in 1736 to join up with Gmelin on The Great Northern Expedition. Amman’s health began to seriously decline in St. Petersburg where he died aged only 34 on 4 December 1741. Before his death, Amman produced an unpublished descriptive list of the plants cultivated in the Academy Botanic Garden. The phrase name for Gmelin’s Siberian lily *Lilium minii colore cruentum* was included in this list as number 863: with the citation of Gmelin and the River Lena (Amman 1739–1740: 41). This name was not included in the published Catalogue of the plants in the garden, but Amman did include two other lilies, also with phrase names, which had been collected by Messerschmidt in Siberia (Amman 1739: 105–106). These lilies, cited later by Gmelin (1747: 43), would eventually be described as *Liliummartagon* L. and *L.pumilum* Redouté. With reference to the latter, he refers to Messerschmidt and the use of Sarana:

138. LILIUM reflexum, montanum, humile, angustifolium, aurantium, Sarana Mungulis in Dauria, Messersschm [138. The low growing, reflexed, orange, mountain lily, Sarana of the Mongolians in Dauria, Messerschmidt] (Amman 1739: 105). In the same text, he also provided additional information that more than one lily was known by the name Sarana, translated from Latin “[….] both these Lilies of Russia should be called Sarana, borrowed from the Tartars; the Tunguts and Burats dig up and eat the bulbs; both lilies come from the plains as well as from the mountains of Dauria” (Amman 1739: 105).

Following Amman’s early death in 1741, the Academy Botanic Garden was placed first under Johann Siegesbeck, then briefly in 1747 to Gmelin and later Krascheninnikof from 1747 to 1749, who was subsequently appointed Professor of the Academy in 1750. The Academy Garden was finally closed in 1812 (Sokoloff et al. 2002: 151). In 1823, the Apothecary Garden became the Imperial Botanical Garden and is now the Vladimir Komarov Botanical Institute’s Botanical Garden of Peter the Great.

## ﻿Herbarium specimens of the Daurian lily

The Academy purchased Amman’s herbarium and drawings in 1743; however, his possessions were already being kept in the Kunstkammer in 1741 at the very end of his life. The bulk of the Kunstkammer Herbarium, including an Herbarium bought by Tsar Peter I in 1717 from Frederik Ruysch and which was compiled by Amman, was largely destroyed by a fire on 5 December 1747 with additional damage to the specimens caused by the water which was used to put the fire out. Amongst the collections that were destroyed were many plants from Messerschmidt, Gmelin and Steller (Sytin 2022: 180). Much of Gmelin’s Herbarium was in fact incorporated into the Kunstkammer in 1757, two years after his death, while many Siberian specimens were sold in 1808 (see below).

Another of Gmelin’s botanical companions in Siberia was Alexander Wilhelm Martini (1702–1781). Martini travelled with Gmelin across much of Siberia from 1740 to 1743 collecting botanical specimens and copying his notes (Sebald 1983: 1). These were deposited in the Herbarium of the Natural History Museum of Stuttgart (Staatliches Museum für Naturkunde, STU). Any material of *L.pensylvanicum* collected by Martini in STU would have been destroyed by bombing during World War Two (Anette Rosenbauer pers. comm.)

A thorough search through the collections of *Lilium* in the Komarov Botanical Institute Herbarium (LE), where any possible specimens of *L.pensylvanicum* collected by Gmelin, Krascheninnikov or Steller would have been deposited, did not yield any such material. It is possible that any specimens collected during the Great Northern Expedition could have been destroyed as a result of the disastrous fire in the Kunstkammer of 1747. There are, however, in the Herbarium still some unopened bundles of specimens from the Kunstkammer collections that have not yet been incorporated into the general collections. Part of Steller’s collections were sold by Pallas at auction and were bought by the British botanist Aylmer Bourke Lambert in 1808 along with Pallas’s own collections, but none of Steller’s specimens of *Lilium* has been located. Lambert’s Herbarium was sold and dispersed after his death in 1842. There is also no lily specimen to be found in BM amongst the Amman collections which Amman had sent to his former employer Sir Hans Sloane.

**Figure 4.**
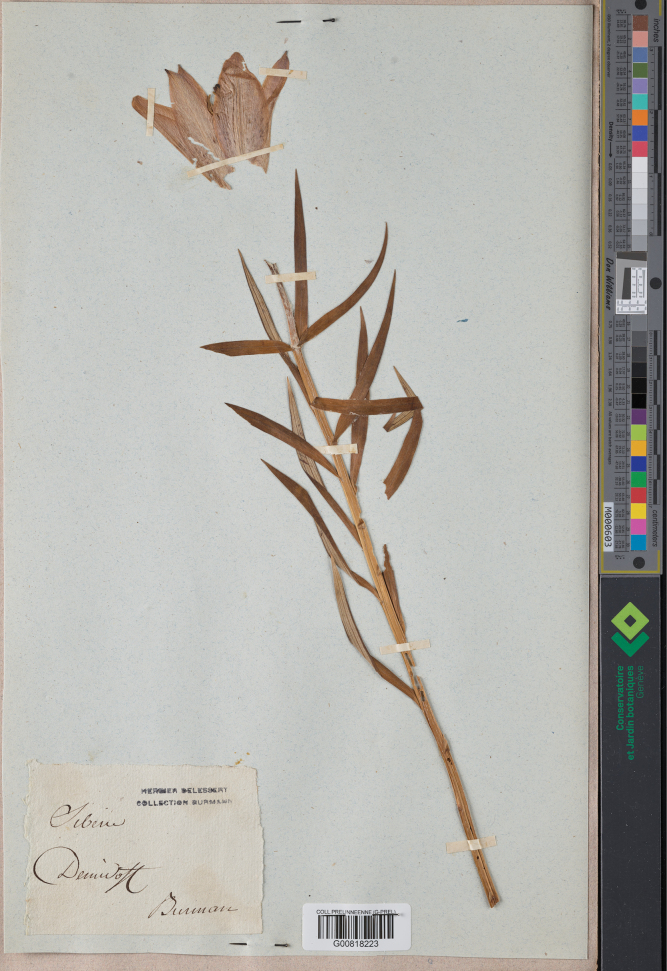
Burman Herbarium specimen G00818223 of *L.pensylvanicum* labelled ‘Siberie Demidoff’.

There is one specimen of interest amongst the pre-Linnaean G-PREL collections of the Geneva Herbarium [G00818223]. This sheet (Fig. 4), listed as SIB 567883/1, clearly represents a single flowering specimen of *L.pensylvanicum*. It has a label with “Herbier Delessert collection Burman” printed in ink and also in ink written on the same label by hand “Siberie Demidoff Burman”. The Herbarium of the Dutch botanists Johannes Burman (1706–1779) and his son Nicolaas Laurens Burman (1734–1793) was acquired in 1810 after the death of the latter’s widow by the French banker and naturalist [Jules Paul] Benjamin Delessert (1773–1847). In 1869, the city of Geneva acquired Delessert’s Herbarium including the Burman Herbarium from his daughters. This specimen of *L.pensylvanicum* possibly originated from one of the collectors on the Great Northern Expedition. There is no date or additional information on the sheet as to which of the Demidov brothers (see below) had acquired the sheet or how the specimen had then been incorporated into the Burman collections. One possibility is that it may have been sent initially by Grigory Demidov to Linnaeus who then distributed it to either Johannes or his son in Amsterdam.

Unconnected with these earlier collections, the German Naturalist Peter Simon Pallas (1741–1811) also visited Siberia having joined the St. Petersburg Academy of Sciences in 1767. Under the instructions of Tsarina Catherine II, Pallas led an expedition for the Academy across Russia from 1768–1774 as far as Lake Baikal. In his Corrigendum (Ker Gawler 1809b: sub t. 1210), Gawler cites a specimen collected by Pallas from this expedition. There is a sheet in BM (Fig. 7) with four collections of the Daurian lily on one sheet, three are in flower and one in fruit [BM000551418]. Three labels on the sheet attest to this having been a collection by Pallas, one has “Liliumbulbiferum spontan. e Dauria’, another has “Herb. Pallas” and the third has “Liliumbulbiferum spontan. Sibir.”. There is however, no evidence that Pallas sent back to St. Petersburg any living plants.

## ﻿The Demidovs, amateur botanists and garden makers

The wealthy salt, iron and steel mining and manufacturing family of Demidov (or Demidoff) created a botanic garden in the village of Krasnoe near Solikamsk, west of the Ural Mountains in ca. 1730 near the family’s salt mines (Sokoloff et al. 2002: 161). Three brothers, Prokofy Akinfievich Demidov (1710–1786), Grigory Akinfievich Demidov (1715–1761) and Nikita Akinfievich Demidov (1724–1787) were all keen amateur botanists involved with the creation of that garden. Prokofy exchanged plants with Amman in St. Petersburg and with the Moscow Apothecary Garden, moving in 1756 to Neskuchny on the Moskva River, which was then just outside Moscow, to start his own private botanic garden. He was later visited by the plant collector Peter Simon Pallas in 1773 who, in 1781, published a catalogue of the plants cultivated there (Sokoloff et al. 2002: 163). The Daurian lily was not included in that catalogue. The palace and garden were sold after his death to Count Alexei Orlov-Chesmensky.

Grigory Demidov remained in Solikamsk where he exchanged plants and corresponded with Amman in St. Petersburg, as well as with Traugott Gerber (1710–1743) who, from 1735, was Director of the Apothecary Garden in Moscow. Grigory was visited by Gerhard Müller and Johann Gmelin in 1743 on their return from Siberia bringing with them their collection of Siberian plants destined for the St. Petersburg Academy Botanic Garden (Elina 2022). Grigory was also visited by Steller and the adjunct ethnographer J. E. Fischer in April 1746, only a few months before Steller’s death in Tyumen on 12 November that year (Elina 2022). Steller who was suffering from illness, stayed for several months having brought with him his large collection of frozen Siberian plants which he planted to defrost in Demidov’s garden for safekeeping. The intention was for the plants to be given a temporary home before being sent on to St. Petersburg. These plants and an herbarium were collected from across Siberia; near the Lena River, around Lake Baikal and near Irkutsk. Following Steller’s death, his collections were eventually sent on to St. Petersburg by Grigory arriving there on 11 March 1748 (Elina 2022). It is clear, however, that many of Steller’s living plants remained in Solikamsk. Having moved to his house fronting the Moyka River in St. Petersburg, leaving the Solikamsk garden in the hands of a reliable gardener, Grigory wrote his first letter to Linnaeus (L0877) on 26 February 1748 (Elina 2022). In this letter, he included 62 packets of seeds of Siberian plants from Steller’s collections, followed on 28 October 1748 (L0969) by ten more sets of seeds (Savage 1945: vii; Elina 2022). It is not known how large his garden was in St. Petersburg, but he was believed to have used it as a temporary repository for growing plants. No specific mention of any lilies was made in these letters. Despite several additional letters in which Steller’s Herbarium was sent to Linnaeus for determining the contents and which were later returned to Grigory (L0915; L0946; L1359), there was no mention of any *Lilium*.

It is still likely that it had been Grigory Demidov who had sent the specimen of *L.pensylvanicum* to the older Burman from Steller’s original Siberian collections (Fig. 4). There are other specimens from Siberia in G that simply bear the label “Demidoff, Siberie” (Martin Callmander, pers. comm.). Many of Grigory Demidov’s living Siberian collections eventually went to his brother Prokofy’s garden at Neskuchny near Moscow after the sale of the Solikamsk estate in 1772 (Elina 2022).

## ﻿How the Daurian lily acquired an American name

**Figure 5.**
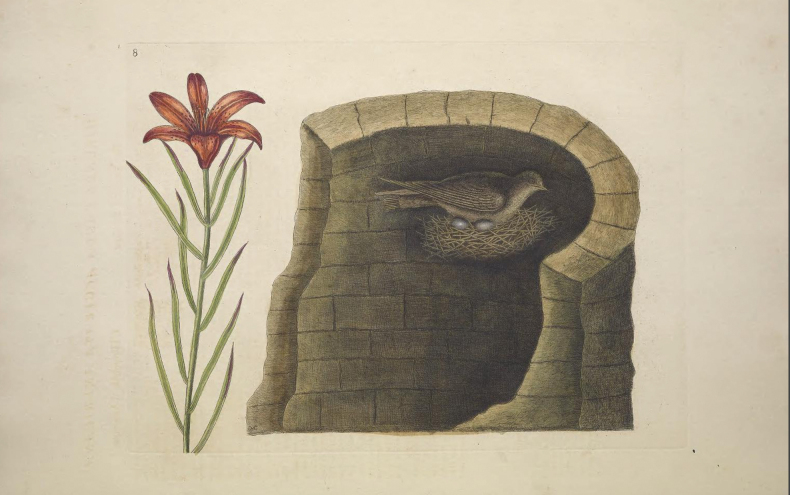
Watercolour of *L.pensylvanicum* as ‘*Liliumangustifolium*, *flore rubro singulari*’ in the Appendix to Mark Catesby’s The Natural History of Carolina, Florida and the Bahama Islands (Catesby 1747: App. t. 8).

After the arrival of the Daurian lily in Europe, it was confused with *L.philadelphicum*, one of the two upwards-facing bowl-shaped lilies from North America. A lily that was believed to have come from North America was painted by Mark Catesby (1683–1749) in the Appendix (Fig. 5) of his The Natural History of Carolina, Florida and the Bahama Islands (Catesby 1747: App. t. 8). The Appendix comprised twenty additional plates prepared after the ten original parts published in two volumes. It is known that, after Catesby’s return to England in 1726 from his own collecting in Carolina, many plants he depicted in the Appendix were sent from John Clayton and John Bartram in North America to either his friend Peter Collinson (1694–1768) or to Catesby himself (Dandy 1958: 111).

Catesby described the lily which he depicted in the Appendix with the name “*Lilium angustifolium, flore rubro singulari*, Le Lys rouge de Pensylvanie” (Catesby 1747: App. t. 8). In an Advertisement for this Appendix (of which only two copies are known to exist), the lily was called *Liliumrubrumminimum* (Overstreet 2015: 164). When these additional plates were presented to the Royal Society, the Society’s Second Secretary and Sir Hans Sloane’s Medical Assistant Cromwell Mortimer, added the statement regarding plate eight of the Appendix: “Lilium angustifolium, flore rubro singulari. The red Pensylvanian Lily. This lily comes from Pensylvania. It agrees with our climate” (Mortimer 1748: 161).

Catesby stated in his text to this eighth plate in the Appendix that the flower consists of six deep scarlet petals spotted with very dark red or purple. He also added that the upper part of the stem and underside of the tepals were roughly hairy and that it was a native of Pennsylvania and that, in 1743, he saw it in flower in the garden of Mr. Collinson in Peckham (Catesby 1747: App. t. 8). Peter Collinson, a rich cloth merchant who traded extensively with North America, had very strong connections with Catesby, being his principal sponsor for the publication of the Natural History and having given him a very substantial interest-free loan. The question concerning the identity of the plant portrayed in this painting is – did Catesby actually portray a North American lily? Catesby referred to the plant unequivocally as the Pennsylvania lily; however, the illustration is undoubtedly one of Catesby’s least convincing portraits (see Fig. 5). The image of the lily and its brief description were eventually published only two years before Catesby’s death at a time when his eyesight was alleged to have been failing, according to Linnaeus’s apostle Pehr Kalm (Nelson 2015: 17).

In 1747, Catesby presented his final illustrations for the Appendix to the Royal Society (Overstreet: 2015: 158). The only North American lily species that Catesby’s description and image could refer to is the species *L.philadelphicum* and it has naturally been identified as that species (Reveal 2015: 341; M. Skinner, pers. comm.). There is, however, a huge “**but**” here - *L.philadelphicum* usually has at least one whorl of leaves subtending the emergence of the peduncle, whereas Catesby’s image has scattered leaves throughout. Although scattered leaves can, however, occasionally occur on young or small plants of *L.philadelphicum*, there is also Catesby’s comment to consider concerning the presence of rough hairs on the upper stem and on the reverse of the tepals. Any such pubescence does not occur on *L.philadelphicum* whose stem, leaves and tepals are glabrous and occasionally glaucous.

There is an herbarium specimen in BM [BM001047104] originally with the unpublished name *Liliumcollinsoniae* written on it. The name, written by an unknown hand, was probably designed to honour Peter Collinson’s wife Mary. This is readily recognised to be the other North American species with upright flowers and has been correctly identified as *Liliumcatesbaei*. It has narrower tepals than those in *L.philadelphicum*, each tepal has longer and narrower claw-like bases and scattered rather than whorled leaves. This sheet is conserved in the Catesby collection within the Sloane Herbarium. Another specimen also of *L.catesbaei* is conserved in OXF [Sher-0708.14] in the Sherard Herbarium number 700 and is labelled simply “Mr Catesby S. Carolina 1723”.

**Figure 6.**
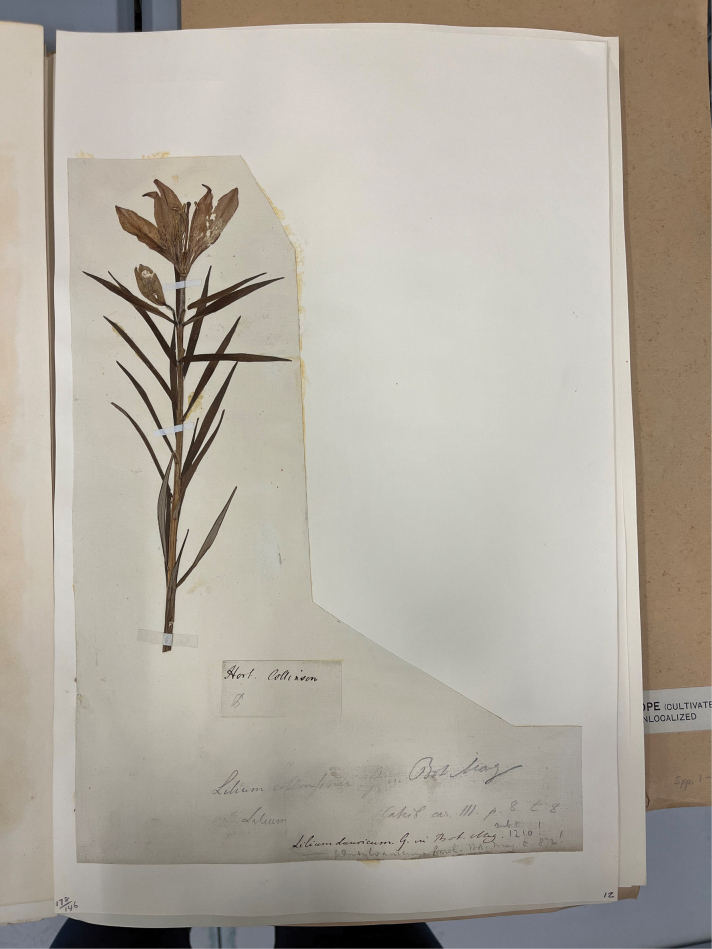
The Hort. Collinson specimen of *L.pensylvanicum* BM014605092.

There is a specimen of *L.pensylvanicum* in BM [BM014605092] with the annotation on one label attached on to the sheet simply with “Hort. Collinson” in ink and with an indecipherable initial in pencil which could be an entwined JG, possibly representing John Gawler (Fig. 6). At the bottom of the sheet are written a succession of references which, reading chronologically are: “Lilium dauricum G. in Bot. Mag. 1210!” in ink with “sub t.” added in pencil above, then immediately under that in pencil “------ pensylvanicum Gawl. Bot. Mag. t. 872!”, the dashed line indicating Lilium from the ref. above it; above that is “Lilium [empty space, possibly with angustifolium, but now rubbed out] Catesby car. III. p. 8. t. 8” in pencil and, at the top, “Lilium collinsonii G. in Bot. Mag.” also in pencil. The specimen has been cut out from its original mount and remounted and consists of an inflorescence with a single upright flower with distinct spotting and an unopened flower bud. There are several narrowly lanceolate leaves scattered along the stem. There is a noticeable presence of pubescence at the junction where the leaves join the stem i.e. in the leaf axils, and along the upper parts of the pedicel and hairs are scattered loosely on the outside of the tepal surfaces. These characters are consistent with Catesby’s description of the species and are to be found on *L.pensylvanicum* i.e. the Daurian lily from east Asia, not on *L.philadelphicum* from North America. There is no date to indicate from which of Collinson’s gardens this specimen was taken, but it could have been the specimen from which Catesby had made his painting.

## ﻿The Daurian lily in Collinson’s catalogue

From the year 1722, Peter Collinson began to compile a catalogue or list of plants growing in his garden in Peckham, which was then merely a village south of London. On the death of his wife’s father in 1749, he inherited Ridgeway House in Mill Hill with a much larger garden of eight acres. There he continued to contribute to his catalogue which, from the start, included his own additional memoranda on the origin and performance of his plants. Many of these memoranda he added either as interleaved additions within his original text or, in some cases, as separate pages. The catalogue remained unpublished during his lifetime. It was, however, published posthumously by the Quaker Lewis Weston Dillwyn (1778–1855) of Sketty Hall, owner of the Cambria Pottery near Swansea, naturalist, abolitionist and Whig politician. Dillwyn basically edited Collinson’s catalogue and rearranged Collinson’s polynomial phrase names into what he perceived to be the correct Linnaean binomials. The named entries listed by Dillwyn as “Not in Catalogue” were largely based on the Linnaean names he had found in Loudon’s Hortus Brittanicus (Loudon 1830; Dillwyn 1843: 1). Moreover, Dillwyn applied to the list of names in Collinson’s catalogue those of Collinson’s memoranda that he surmised to be correctly associated with them (Dillwyn 1843: vi). Many relevant dates of introduction into Collinson’s garden were included in Collinson’s catalogue and subsequent memoranda. Thus, *Liliumphiladelphicum*, according to Collinson, was introduced into the garden in 1730, *L.pensylvanicum* in 1740 and *L.carolinianum* (i.e. *L.catesbaei*) before 1743 and the Siberian scarlet-flowered turkscap *L.pumilum* in 1748 (Dillwyn 1843: vi). In our context, Dillwyn’s identifications based on Collinson’s catalogue and memoranda beg the question as to what Collinson’s *L.pensylvanicum* might have been?

Dillwyn included what he believed to have been *Liliumphiladelphicum* with the statement “Not in Catalogue” adding Collinson’s memorandum: “*1730, June 16, J. Bartram sent me some very elegant red lily roots, flowered in 1740, it rises a foot high; the leaves set round the joints of the stem in tiers, one above another; the flower is the smallest of all the Lilies that I have seen; it consists of six leaves, set wide from each other, of a deep fire or flame colour; one half of the leaf or petal clear, the other half spotted, with very large deep purple brownish spots; one single flower on a stalk, but in the year 1746 it had two flowers from one stalk; from Pensylvania*” (Dillwyn 1843: 30). It is clear that Collinson is referring to *L.philadelphicum* sent to him by John Bartram and that Dillwyn had correctly identified this species.

Another lily which Dillwyn attributed to “L.pensylvanicum of Bot. Mag.” was mentioned in Collinson’s catalogue with the phrase and reference “Lilium acadiense pumilum flore rubro punctato. Dodart’s Mem.” (Dillwyn 1843: 29). Collinson’s reference is to the phrase name in the work of Dionys Dodart with illustrations by Nicolas Robert (Dodart 1686: 91). Dodart, in turn, stated that the Acadian lily was sent from Cayenne by Monsieur Richer of the Académie Royale des Sciences. This would have been Jean Richer who visited Canada [Acadia] on an astronomical expedition in 1670 for the French Académie Française and who went, in 1672, on a similar expedition to French Guyana. Collinson’s two memoranda referring to this lily are: “*1740. Received from J. Bartram a new Orange Lily, with hoariness on flower and leaves, figured by Catesby*”. The second memorandum read: “*1750, a Pensylvanian Lily, that bears but one flower, dotted with purple, is well described in Dodart’s Memoire des Plantes, and well figured. Since, I have raised many crimson coloured Martagons from Pensylvania seed; all new species*”. The description by Dodart and the illustration by the French royal painter Robert is without doubt also that of *L.philadelphicum*. This lily then was clearly Dillwyn’s “L.pensylvanicum of Bot. Mag.” (Dillwyn 1843: 29), but the statement “hoariness of flower and leaves” is not correct for *L.philadelphicum*, whose parts are glabrous.

Collinson was obviously very keen on the genus *Lilium* as he grew 18 more lilies listed in his catalogue. He included as postscripts various memoranda relating to them. The majority of these lilies are not relevant for this paper, but at least one clearly is. Dillwyn listed “*Liliumpomponium* Var.?” Collinson had written “*Lilium purpuro-croceum majus an floris rubro lutei. Act. Nat. iii., p. 155; this orange or red Lily I raised in plenty, sent me by Dr. Amman, of Petersburgh, sent from Siberia, and the roots are there eat(en) for bread*” (Dillwyn 1843: 30). The descriptive phrase name reflects the name that was used for *L.bulbiferum* by Casper Bauhin in his *Pinax* i.e. “*Liliumpurpurocroceummajus*” (Bauhin 1623: 76). As we have already seen, the Siberian lily is very closely related to the European *L.bulbiferum* and the Siberians’ use of eating sarana polyvega has already been discussed above.

The reference which Collinson gave i.e. Act. Nat. iii p. 155, refers to the same paper by Henckel (Henckel 1733: 354) as that which was also cited by Gmelin (Gmelin 1747: 41), although the pagination of 155 should have been 355 (Jacek Wajer, pers. comm.). As discussed above, this species was introduced from Siberia by 1733 at the latest and it seems likely that seeds or even bulbs may have been cultivated by Amman in St. Petersburg before being sent on to Collinson in Peckham. It would strongly suggest that Collinson’s “Liliumpomponium var.?” was indeed the Daurian lily i.e. *L.pensylvanicum* Ker Gawl.

Why it was that Dillwyn had doubtfully suggested this could be a variety of the western Mediterranean turkscap *Liliumpomponium* L. with its scarlet, highly recurved tepals is possibly explained by his confusing it with the Siberian *L.pumilum*, which does, indeed, have pendent, scarlet flowers with reflexed segments like the European species. Collinson’s description, however, is almost certainly of *L.pensylvanicum* not *L.pumilum* as the latter bears no similarities with Bauhin’s *Liliumpurpurocroceum*.

Immediately above this entry, Dillwyn had listed another lily also with the name *Liliumpomponium* Var.? He attributed to it Collinson’s memorandum “*Lilium Martagon sibiricum, petalium quasi fistalosum flore purpureo nigricante; sent from Moscow*”. Collinson’s memorandum also stated “*1756 sent to me by Mr Demidoff, proprietor of the Siberian Iron Mines, some roots of Siberian Martagon; flowered for the first time May 24 1756; the flower is but little reflexed, and is, I think, the nearest black of any flower I know*” (Dillwyn 1843: 30). This, according to the description, refers to *Fritillariacamschatcensis* (L.) Ker-Gawl., a species with deep reddish-black flowers from east Siberia and which also occurs across the Bering Strait in Alaska and the western USA. This was evidently cultivated much later in Collinson’s garden in Mill Hill. Linnaeus had originally called this species *Liliumcamschatcense* L. (Linnaeus 1753: 303).

Dillwyn had added two further entries which are somewhat confusing. The first simply is *L.pumilum* with Collinson’s phrase “Lilium sibiricum pumilum cruente”. Collinson’s memorandum added “*1748, July, flowered orange or yellow lily, I raised seed from Daurica, called Saranna; can perceive very little difference from those we had before, except they grow not so high, or produce so many flowers”*. Then another memorandum “*Called Sarana by the Tartars; they dry and powder the roots, and mix for bread in their broths, for they grow no corn*” (Dillwyn 1843: 30).

This appears to be another collection of *L.pensylvanicum* which was identified incorrectly as *L.pumilum*. Both these lilies, as well as *Fritillariacamschatcensis*, are known under the name Sarana and all three species have been used as food in Siberia (Ståhlberg and Svanberg 2006). The second entry has “*Lilium sibiricum pumilum novum, flore rubro nigro quatuor unciarum altitudinem, new*” [A new Siberian lily, four inches high, with reddish-black flowers]. This is probably only a variant of *Fritillariacamschatcensis*.

As Amman had died in 1741, Collinson’s memorandum on the lily that he described as *Lilium purpuro-croceum majus an floris rubro lutei* reveals that Amman did, indeed, already have the Siberian lily now known as *L.pensylvanicum* in cultivation well before that date. It is also possible that he was cultivating the lily shortly after 1733 when it may have arrived in Germany. The Collinson herbarium specimen now in BM [BM014605092] with distinct pubescence in its upper parts might have originated from one of the plants sent by Amman to Collinson and which was subsequently painted by Catesby (Catesby 1747: App. t. 8). There is, however, no indication of a date on the sheet and it may have been from a later introduction. Nor is there any indication by Collinson as to whether he grew the lily in his garden in Peckham or later in the larger one in Mill Hill of eight acres that he moved into in 1749, thereby ruling out Catesby’s involvement.

## ﻿Ker Gawler’s *Liliumpensylvanicum*

The amateur botanist John Bellenden Ker Gawler, later Bellenden Ker (1764–1842 cited here as Ker Gawler), having seen Catesby’s illustration (Fig. 5) and description, unwittingly described the Siberian lily with an illustration under the name *Liliumpensylvanicum* Ker Gawl. (Fig. 1), citing Catesby’s Appendix (as volume three of his Natural History) as a reference (Ker Gawler 1805: t. 872). Ker Gawler clearly believed that he was giving Catesby’s lily from North America a valid binomial; however, the illustration painted by Sydenham Edwards and the description without doubt represents the Asian species. The lanate peduncle, outside of the corolla “floccoso-lanata” and pubescence at the junction of the leaves with the stem and along the leaf margins are not characters found on the North American species (Mark Skinner, pers. comm.). As Ker Gawler himself admitted “The only mention of this species that we have been able to find, is in the above quoted work of Catesby” (Ker Gawler 1805: t. 872 text), adding that it had flowered in Peter Collinson’s garden in 1745 and that a specimen from that collection was deposited in the Banksian Herbarium [now in the General Herbarium BM]. Additionally, of note is Ker Gawler’s comment “The affinity with *Liliumbulbiferum* is so great that we can hardly bring ourselves to consider it specifically distinct” (Ker Gawler 1805: t. 872 text). Ker Gawler mentioned that the four or five upper leaves are whorled, a character found sometimes in the Asiatic species and frequently *L.philadelphicum*, but he went on to say that the bulb sends out numerous creeping shoots, a character typical of the Asian plants, but not of *L.philadelphicum*.

The illustration accompanying the written description, however, was prepared by Sydenham Edwards from a plant cultivated by the London nursery of Whitley & Brames of Old Brompton and was dated 1 September 1805 and published by the late William Curtis’s brother Thomas in the Botanical Magazine. The large nursery of Whitley & Brames covering eight acres next to Gloucester Road and Old Brompton Road was founded in 1784 by Frank Thoburn. Between 1801 and 1810, it was run by partners Reginald Whitley (1754–1835) and Peter Brames (? -1834), specialising in hardy herbaceous and alpine plants (Harvey 1973: 186). The error that Ker Gawler made as to the origin of the lily was compounded by the fact that one of the proprietors of the nursery had informed Ker Gawler that the plant in the nursery had also come originally from North America. Nowhere in Ker Gawler’s text is the name of the provider of the lily to the nursery mentioned.

Could the provider of the Daurian lily to Whitley and Brames have been the botanist Richard Anthony Salisbury (1761–1829)? Salisbury was one of the founding members in 1804 of the Horticultural Society of London [later RHS], but was also the Society’s first secretary from 1805 to 1816. After Peter Collinson’s death in 1768, Ridgeway House passed to his son Michael, another amateur botanist, who died in 1795. Michael’s son Charles Streynsham Collinson sold Ridgeway House and its fine garden to R. A. Salisbury in 1801. Salisbury lived at Ridgeway House until 1806 and had befriended the Burchell family, nurserymen of Kings Road, Fulham. In his will he left the bulk of his estate to the son of Matthew Burchell, the South African explorer William John Burchell (1781–1863). The Burchell nursery was acquired in 1810 by Reginald Whitley, Peter Brames and Thomas Milne. In the absence of any nursery records of the time, it is not beyond the realms of possibility that Salisbury could have grown the Daurian lily from Collinson’s collection and that either Whitley or Brames had acquired it from him for Ker Gawler to describe and Sydenham Edwards to paint.

### ﻿First doubts on the lily’s origin

Ker Gawler cast doubt on his own assertion that the illustration and description of his *L.pensylvanicum* originated from North America three years later. In a discussion under his depiction of *Pancratiumrotatum* Ker Gawl. [now *Hymenocallisrotata* (Ker Gawl.) Herb.], in which he propounded correctly that Linnaeus had erred in his identification of *Pancratiumcarolinianum* L., mistakenly as a North American species instead of the European *P.maritimum* L., he again discussed *Liliumpensylvanicum* (Ker Gawler 1808: t. 1082). In that work, he stated that he believed the lily’s origin was not from North America, but possibly from China or Japan and that the lily was likely to be the same as what Thunberg had called either *L.philadelphicum* or *L.bulbiferum* from Japan. In neither case was Thunberg referring to either *L.philadelphicum* L. or *L.bulbiferum* L. (Ker Gawler 1808: t. 1082).

Ker Gawler stated the following year, after his diagnosis of the differences between *L.pensylvanicum* and his depiction and description of *L.concolor* (Ker Gawler 1809a: t. 1165), that he still needed to ascertain the country of origin of his original description of *L.pensylvanicum*. He added too that he had yet to establish how it differed from *L.bulbiferum* and that Reginald Whitley, upon reflection, believed it to have been a plant from Russia. Ker Gawler continued to fan the flames of confusion by stating that perhaps Catesby had only guessed that the plant growing in Peter Collinson’s garden and which Catesby had painted (Fig. 5) was from North America, inferring that it was the same as that figured by Ker Gawler. Crucially though, Ker Gawler then posited two questions – was this the variety 2 foliis angustioribus – (α) flore miniato of the *Liliumbulbiferum* of Gmelin’s Flora Sibirica; and a Siberian plant? Or was it from China? He stated categorically, however, that it was not a native of North America (Ker Gawler 1809a: t. 1165).

### ﻿The Corrigendum and renaming

Ker Gawler finally added a Corrigendum later that year in which he attempted to rename *L.pensylvanicum* Ker Gawl. as *L.dauricum* Ker Gawl. (Ker Gawler 1809b: sub t. 1210). His Corrigendum read: “No. 872. For Liliumpensylvanicum. Pensylvanian Lily, read Liliumdauricum, Siberian Lily”. Ker Gawler renamed the lily after the land of the Daur peoples in Dauria or Dahuria, a mountainous region once located near Lake Baikal encompassing modern-day Transbaikal and comprising Buryatia, Zabaykalsky Krai and the Amur River region. This renaming has, of course, subsequently engendered a great deal of confusion and has subsequently been formally rejected.

In Ker Gawler’s protologue for *L.dauricum* (Ker Gawler 1809b: sub t. 1210), he included three synonyms:

Liliumbulbiferum. Pallas. Herb. penes Dom. A. B. Lambert

L[ilium]. 2 foliis angustioribus (α) flore miniato Gmel. Sib. 1. 41

L[ilium]. angustifolium flore rubro singulari. Catesby Carol. 3 p. 8. t. 8. false ab auctore pro Americae indigena datum: tabula a planta in Horto Londini suburbano florida desumpta fuit. [Narrow-leaved lily with a single red flower in Catesby’s Carol. 3, page 8 t. 8 falsely provided by the author as a native American plant: the illustration was made from a flowering plant in a garden in the suburbs of London].

**Figure 7.**
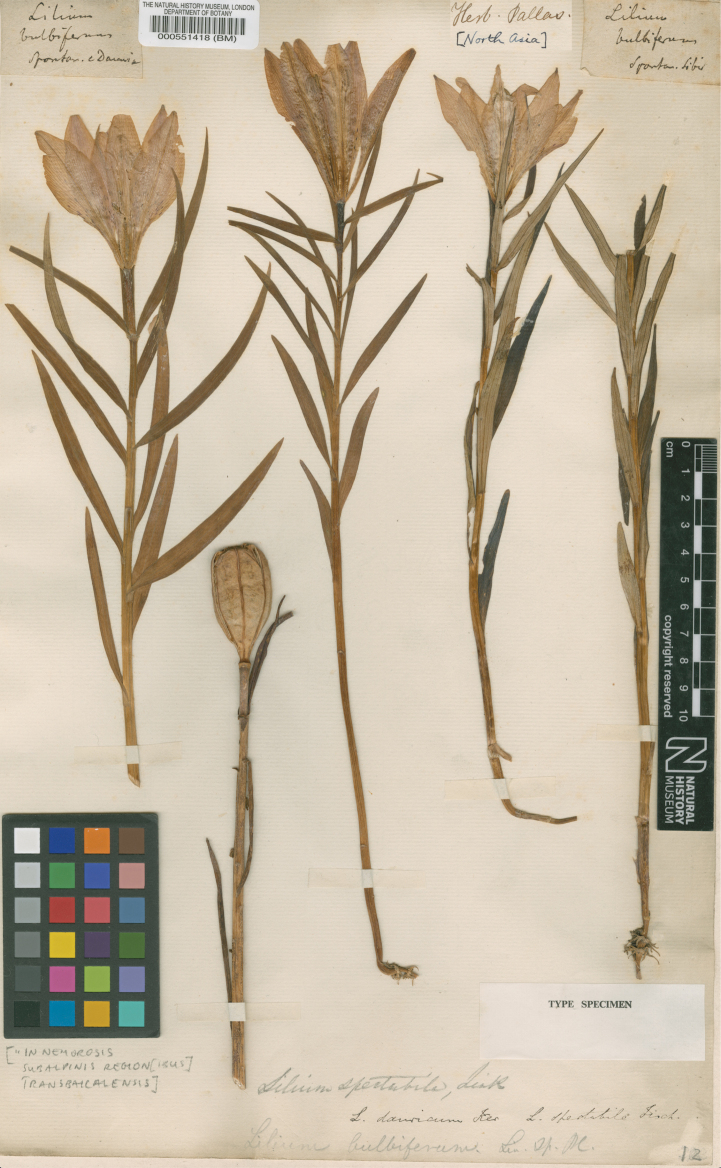
Peter Simon Pallas’s specimens of *L.pensylvanicum* from Siberia that may have been in Aylmer Bourke Lambert’s Herbarium BM000551418.

Ker Gawler added the statement “In Pallas’s Herbarium at Mr. A. B. Lambert’s, there are several very perfect specimens of the species, gathered in the eastern parts of Siberia” (Ker Gawler 1809b: sub t. 1210). The specimens collected by Pallas in BM [BM000551418] have already been discussed above (Fig. 7). These specimens may well have been at one time in Aylmer Bourke Lambert’s Herbarium as they do, indeed, perfectly represent the Siberian species; however, even though Ker Gawler attempted to change the name of the species to *L.dauricum*, they were not included in the original protologue. Only material cited within the protologue of *L.pensylvanicum*, however, would be eligible as type (Art. 7.4, Turland et al. 2018). The available elements for typification, therefore, are Edwards’s illustration which accompanies the description, the Catesby illustration t. 8 in his Appendix discussed above and a specimen in the Banksian Herbarium in BM (Ker Gawler 1805: t. 872). The illustration accompanying the protologue (Ker Gawler 1805: t. 872) was chosen by Y-D. Gao in Taxon 70(5): 1139 (2021) as the lectotype.

### ﻿Ker Gawler’s conclusion

Ker Gawler later reiterated his conclusion that *L.pensylvanicum* was, indeed, from Siberia in a note under the description of the North American lily L.philadelphicumvar.andinum (Nutt.) Ker Gawl. (Ker Gawler 1821: sub. t. 594). It is worth reiterating his note verbatim for clarity:

“*Owing to a mistake originating with Catesby, a species of this genus is given by Messrs. Pursh and Nuttall to America, while in fact it does not belong to that quarter of the globe*. Liliumangustifolium flore rubro singulari *of the Natural History of Carolina was described and figured from a plant in Mr. Peter Collinson’s garden at Peckham, and being conceived in the recollection of Catesby to be the same with one he had seen in America, was published by him in the above History as such*. *A sample of that plant from the same garden is also preserved in the Banksian Herbarium. Many years after it was published by ourselves in Curtis’s Magazine (No. 872), under the title of*L.pensylvanicum, *upon this authority; but having subsequently detected the mistake, we corrected it in No. 1210 (over-leaf) of the same work; where we republished the species by the name of*L.dauricum, *having ascertained its Siberian origin from native samples in the Lambertian Herbarium*. *This emendation however, having been overlooked in the works of Messrs. Pursh and Nuttall, as well as in the Hortus Kewensis, it may not be useless to restate the whole correction. The species Catesby mistook it for was probably* Lilium Catesbaei, *if not* philadelphicum” (Ker Gawler 1821: sub t. 594).

Ker Gawler added under his list of synonyms another short note: “*Mr. Nuttall seems to have been puzzled in adopting the plant as American; and suggests the possibility of its being a hybrid produced during culture, because of its occasionally wanting the pistil in our gardens; an effect more probably of luxuriance, as the pistil is usually perfect with us and frequently fertile. The species is in fact, very close to* bulbiferum, *but we believe it nevertheless to be truly distinct*” (Ker Gawler 1821: sub. t. 594).

## ﻿Taxonomic treatment

The distribution of *L.pensylvanicum* covers a large area in eastern Asia and, as such, also encompasses some morphological variation mainly in differences of stem stature, flower colour and leaf width. These might best be regarded as of horticultural rather than botanical significance as evidenced in the synonymy. The significantly shorter stature and narrower leaf width of the alpine forms of this species found in Sakhalin Island and in alpine meadows of the Amur Region and the Primorye Mountains we believe merit varietal recognition. We have, therefore, considered that the typical taller more robust, wider leaved variety and the alpine variety are worthy of taxonomic recognition.

### ﻿Species description for *Liliumpensylvanicum*

**Description. *Bulb*** ovoid-globose, 2–4 × 2–4 cm with numerous white, fleshy, convex, lanceolate, acute scales, stoloniferous bearing small subterranean bulbils; ***stems*** (5 –) 30–75 (– 120) cm tall, green, sometimes purple spotted, more or less ribbed, partly or entirely covered with white floccose pubescence especially in upper leaf axils and along inflorescence axis; ***leaves*** scattered, sessile, blades linear-lanceolate to lanceolate, 1–3 (– 5) veined, 4–10 × 0.2–2 cm, apex acuminate, margins entire or finely papillose; ***inflorescence*** 1–3 (– 6) flowered, buds frequently covered in floccose hairs; ***pedicels*** erect 1–9 cm long, frequently covered in floccose hairs; ***flowers*** erect, red, orange, rarely yellow, spotted or unspotted with dark brown spots, more or less openly campanulate, perianth of six tepals, obovate-spathulate to oblanceolate 3–6 (– 8) × 1–3 cm, inner three equal in size and shape to outer three, all narrowing to a claw-like base, tepals gently recurving at apex, nectariferous sinus densely pubescent along margins; ***stamens*** shorter than tepals, ***filaments*** glabrous, reddish, anthers 1 cm long, pollen red; ***pistil*** slightly longer than filaments, stigma capitate, 3–lobed; capsule oblong-ovoid, obtusely angled 4–5 × 2–3 cm.

**Habitat.** Forest clearings, meadows, riverbanks and sandy areas.

### ﻿Key to the varieties of *Liliumpensylvanicum*

**Table:** 

1	Stems 30–75 (–120) cm; leaves 3–5 veined, lanceolate, 6–10 × 0.5–2 cm; perianth segments 6–9 cm long	**L.var.pensylvanicum**
–	Stems (5–) 10–20 cm; leaves 1–3 veined, linear-lanceolate, 4–5 × 0.2–0.5 cm; perianth segments 5–6 cm long	**L.var.alpinum**

#### ﻿ Lilium pensylvanicum var. pensylvanicum

Taxon classificationPlantaeLilialesLiliaceae

Ker Gawl., Bot. Mag. 22 t. 872 (1805). Lectotype designated by Y-D Gao in Taxon 70(5): 1139 (2021) [icon] Bot. Mag. 22 t. 872 (1805)

A8B0BB65-D38E-5026-95C8-1F29EAA41123

≡ L.dauricum Ker Gawl. Bot. Mag. 30 corrigendum sub t. 1210 (1809).
≡ L.bulbiferumsubsp.davuricum Baker, Gard. Chron. 1871(2): 1034 (1871).
≡ L.maculatumsubsp.davuricum (Baker) H.Hara, J. Jap. Bot. 38(8): 249 (1963).
≡ L.maculatumvar.davuricum (Baker) Ohwi, Fl. Japan: 297 (1965).
= Liliumdauricum [as L.davuricum] var.tigrinum Regel, Gartenfl. 21: 295 (1872) no ref. or type cited, merely the statement “compare with Gawler t. 872”.
= Liliumdauricum [as *davuricum*] var.costatum Regel, Gartenfl. 21: 295 (1872) no. ref. or type cited, merely the statement “compare with Gawler t. 872”.
= Liliumpseudodahuricum M.Fedoss. & S.Fedoss., Acta Comment. Imp. Univ. Jurjev. 7(2) Delectus Plantarum Exsiccatarum: 45 (1899). Type: *Hubelmann 50* (TU) [Tartu, Estonia] not located. ***Lectotype*** designated here: Russia, Chitinskaya Oblast, Dauriya, forest meadows and thickets around the city of Nerchinsk, 20 June 1898, *M.Gubelmann 50* (lecto. KFTA!) [KFTA0003266].
= Liliumsachalinense Vrishcz, Novosti Sist. Vyssh. Rast. 5: 48 (1968). ***Holotype***: Russia Far East, Sachalin [Sakhalin], “litus occidentale, prope opp. Alexandrovsk, western coast between Niarmi and Chirkumnay. 22 June 1916, *O.A.Derbek* s.n. (holo. LE!) [LE01010710].
= Liliumpensylvanicumf.praecox Vrishcz, Spisok Rast. Gerb. Fl. S.S.S.R. Bot. Inst. Vsesoyuzn. Akad. Nauk 18(90–102): 38. (1970) ***Lectotype*** designated here from isotypes: Russia, Siberia, Promorje Prov. [Primorsky Krai] Anuchinsky distr. In pratis varie herbosis 12 June 1967, *D.L.Vrishcz 3104* ex Fl. SSSR 4961(lecto. LE!) [LE01075423]; isolectotypes: (BM!) [BM013719281]; (DAO!) [DAO000466238]; (E!) [E01184390]; (ERE) [ERE0004422]; (JE!) [JE00009931]; (L!) [L1451491]; (L!) [L1451492]; (LE!) [LE01075422]; (MO!) [MO3459011]; (MW!) [MW0043937]; (PE!) [PE01713340]; (TK).

##### Note.

Dina Lukinichna Vrishcz did not designate a holotype for the name L.pensylvanicumf.praecox and we, therefore, designate a lectotype here from an isotype in LE.

- *Liliumsibiricum* Willd., Enum. Pl. Hort. Berol., Suppl.: 17 (1814), nom. nud.
- *Liliumdahuricum* Reuthe, Gartenflora 40: 476 (1891), nom. nud.

##### Distribution.

**China** – Hebei, Heilongjiang, Jilin, Liaoning, Nei Mongol; **Japan** – Hokkaido, north Honshu; **Mongolia**; **North Korea**; **Russia** – Amur, Buryatia, Irkutsk, Kamchatka, Khabarovsk, Krasnoyarsk, Kuril Islands, Primorye, Sakhalin, Yakutskiya, Zabaykalsky; **South Korea.**

##### Phenology.

Flowering period is from June to July.

#### ﻿ Lilium pensylvanicum var. alpinum

Taxon classificationPlantaeLilialesLiliaceae

(N.I.Kuznetsov) J.Compton & Sytin comb. nov.

7C280D5E-049E-5324-92DC-69CCDEECAC7B

urn:lsid:ipni.org:names:77333277-1

##### Basionym.

**Figure 8.**
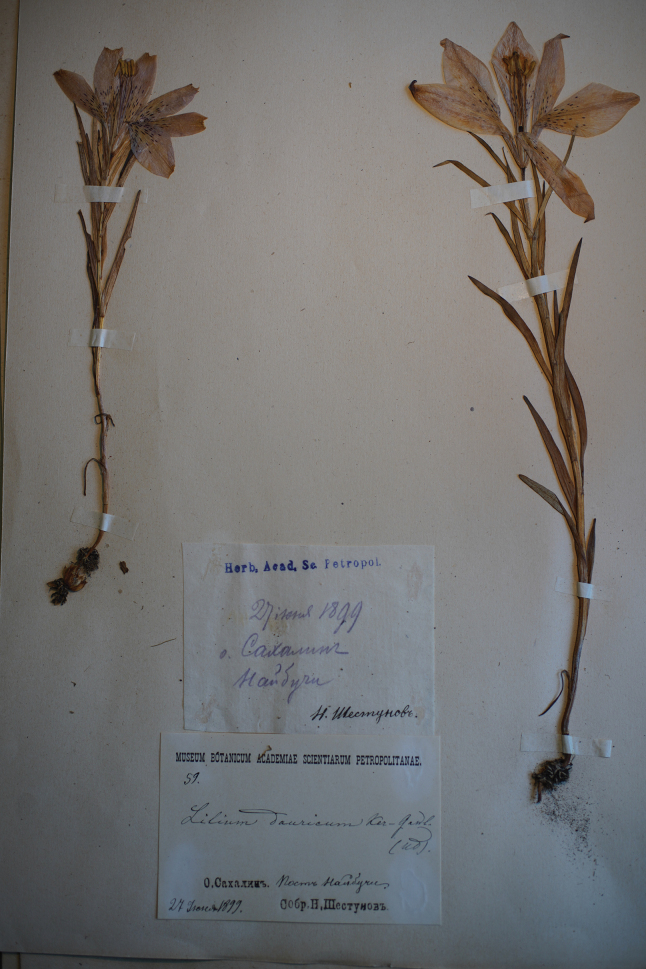
Lectotype specimen *N.Shestunov 59* of Liliumdauricumvar.alpinum LE01075424 designated by Compton & Sytin, this paper.

Liliumdauricumvar.alpinum N.I.Kuznetsov, Trudy Bot. Muz. Rosiisk. Akad. Nauk 18: 80 (1920). ***Lectotype*** designated here from syntypes (Fig. 8): Russia Far East, Sakhalin Ostrov, Naibuchi Post, [near Dolinsk], 27 June 1899, *N.Shestunov 59* (lecto. LE!) [LE01075424]; syntypes: Russia Far East, Amurskaya Oblast, Chamberlain N. L. Gondatti Expedition, railway construction line between Nevers and Urusha stations, loam deposits on river bank, 1 May 1910, *Kvashnin-Samarin 204* (LE!) [LE01075428]; Russia Far East, Amur Oblast, upper reaches of the River Rakindi [Reka Pravaya Rakindi], no collector cited (n.v.); Russia Far East, Sakhalin Ostrov, among rocky mountains, near Due [Dui], 30 May 1872, [*Foma* or *Tomash, Matveyevich*] *Avgustinovich s.n.* (n.v.); Russia Far East, Primoriye Region [Primorsky Krai], Udskoi, [Udskoye, now Khabarovsk Krai] *P.Meyer s.n.* (n.v.).

##### Distribution.

**Russia** – Amur, Khabarovsk, Primorye, Sakhalin.

This variety probably has a wider distribution than indicated from Herbarium material seen in LE. It is, for example, likely to include short plants known informally as “rebunense” from Rebun Island off the north-west corner of Hokkaido, Japan.

##### Phenology.

Flowering period is from May to June.

## Supplementary Material

XML Treatment for Lilium pensylvanicum var. pensylvanicum

XML Treatment for Lilium pensylvanicum var. alpinum

## ﻿Additional information

### Author ORCIDs

James A. Compton https://orcid.org/0000-0002-5421-1554

### Data availability

All of the data that support the findings of this study are available in the main text.
